# Scrapie-Responsive Gene 1 Promotes Chondrogenic Differentiation of Umbilical Cord Mesenchymal Stem Cells via Wnt5a

**DOI:** 10.1155/2022/9124277

**Published:** 2022-01-27

**Authors:** Yuchuan Zhou, Tian Zheng, Lin Li, Yangfang Guo, Jiayu Xiao, Jin Wang, Zhiqin Du, Hui Gao, Weiwei Tang, Liusan Yang, Haiyan Hu, Xiaodan Wang, Mingyao Meng, Zongliu Hou

**Affiliations:** ^1^Central Laboratory of Yan'an Hospital Affiliated to Kunming Medical University, Kunming, China; ^2^Kunming Medical University, Kunming, China; ^3^Key Laboratory of Tumor Immunological Prevention and Treatment of Yunnan Province, Kunming, China; ^4^Yunnan Cell Biology and Clinical Translation Research Center, Kunming, China; ^5^Orthopaedics Department of Yan'an Hospital Affiliated to Kunming Medical University, Kunming, China; ^6^Radiology Department of Yan'an Hospital Affiliated to Kunming Medical University, Kunming, China

## Abstract

**Objective:**

Repair of cartilage defects, a common condition resulting from many factors, is still a great challenge. Based on their chondrogenic differentiation ability, mesenchymal stem cell- (MSC-) based cartilage regeneration is a promising approach for cartilage defect repair. However, MSC differentiation into chondroblasts or related cell lineages is elaborately controlled by stem cell differentiation stage factors and affected by an array of bioactive elements, which may impede the efficient production of target cells. Thus, identifying a single transcription factor to promote chondrogenic differentiation is critical. Herein, we explored the mechanism by which scrapie-responsive gene 1 (*SCRG1*), a candidate gene for cartilage regeneration promotion, regulates chondrogenic differentiation of MSCs.

**Methods:**

Expression of *SCRG1* was detected in umbilical cord-derived MSCs (UCMSCs) by quantitative reverse transcription polymerase chain reaction (qRT-PCR) and immunohistochemical analysis during chondrogenic differentiation. The function of SCRG1 in chondrogenic potential was evaluated after gene knockdown or overexpression by lentiviral vectors. Finally, a rabbit cartilage defect model was established to evaluate the effect of SCRG1 on cartilage repair *in vivo*.

**Results:**

Expression of SCRG1 was upregulated during *in vitro* chondrogenic differentiation of UCMSCs. *SCRG1* knockdown inhibited chondrogenic differentiation of UCMSCs, while *SCRG1* overexpression promoted chondrogenic differentiation of UCMSCs *in vitro.* In addition, UCMSC overexpressing SCRG1 promoted cartilage repair *in vivo*. Mechanistically, SCRG1 promoted chondrogenic differentiation via upregulation of Wnt5a expression and subsequent inhibition of *β*-catenin.

**Conclusion:**

Our results showed that *SCRG1* promotes chondrogenic differentiation of UCMSCs by inhibiting canonical Wnt/*β*-catenin signaling through Wnt5a. Our findings provide a future target for chondrogenic differentiation and cartilage regeneration.

## 1. Introduction

Cartilage defects are usually caused by fracture trauma, surgery, tumor, and other factors. Due to its weak regenerative ability, once cartilage is damaged, the prognosis is poor [1]. At present, cartilage transplantation is considered the most effective form of cartilage repair, and autogenous nonarticular cartilage and mesenchymal stem cell- (MSC-) based tissue-engineered cartilage are the two most important sources of tissue for transplantation. However, there are many problems with autogenous nonarticular cartilage transplantation, including secondary damage, ethical considerations, and insufficient sources, which impede clinical application. In contrast, MSC-based regenerative medicine can overcome many of these issues as MSCs are easy to obtain, exhibit good histocompatibility, and possess biological activity [2]. Thus, MSC-based tissue engineering is a promising alternative therapeutic strategy for cartilage defect repair.

MSCs exhibit self-renewal and multilineage differentiation capability and can differentiate into chondrocytes, osteocytes, adipocytes, muscle cells, neurocytes, and endometrial cells [3, 4]. Somatic umbilical cord MSCs (UCMSCs) have several advantages over other MSCs (e.g., bone marrow and adipose-derived MSCs), such as painless collection, no tumorigenicity, low immunogenicity, and powerful proliferation and differentiation [5, 6]. Thus, UCMSCs are an important source of seed cells for tissue engineering and regenerative medicine. MSC-based tissue engineering involves three closely related components: i.e., MSCs, inducing factors, and scaffolds [7]. Among these components, MSCs, which generate chondrocytes under inducing factors activity, form the foundation for feasible tissue-engineered regeneration. However, the mechanism by which stem cells differentiate into chondrocytes is complex and poorly understood. Currently, chondrogenic differentiation of MSCs is achieved *in vitro* by adding biological inducers such as transforming growth factor *β*, insulin, and glucocorticoids [8, 9]. Several genes are known to be important during chondrogenic differentiation, especially the SRY-box family of transcription factor 9 (SOX9) [10, 11]. Sox9, which is upregulated during chondrogenic differentiation, can increase the expression of chondrogenic genes, such as collagen type II alpha 1 chain (*COL2A1*), and aggrecan (*ACAN*) [12]. Notably, the procedure requires 3–4 weeks for chondrogenic differentiation, which is not convenient in clinical application [13]. Thus, the development of novel methods or targets should help to promote chondrogenic differentiation and improve cartilage regeneration efficacy.

Various bioactive factors promote the differentiation of stem cells and are thus beneficial for tissue regeneration [14]. However, many of these factors remain to be fully explored. In the current study, we aimed to identify and characterize a molecule that may promote chondrogenic differentiation of UCMSCs. Previous transcriptome profiling identified several differentially expressed genes (DEGs) associated with chondrogenic differentiation of MSCs, including the up-regulation of *Scrapie-Responsive Gene 1* (*SCRG1*) [15]. *SCRG1* was first discovered at elevated levels in transmissible spongiform encephalopathies caused by prion infection [16]. *SCRG1* is located on 4q34.1 and encodes a protein 98 amino acids. It is highly expressed and secreted in the nervous system, aorta, and testes [16]. SCRG1 can stimulate chondrogenesis of murine C3H10T1/2 cells (a type of mouse embryonic fibroblast and can be differentiated into chondrocyte) *in vitro* [9]. To date, however, its effects on the chondrogenic differentiation of UCMSCs and the underlying mechanism remain unclear. Here, we explored the mechanism by which SCRG1 participates in chondrogenic differentiation, which should provide new insights into regenerative medicine for cartilage repair.

A series of signaling pathways are involved in cell differentiation, including the Wnt/*β*-catenin, Notch, Indian Hedgehog, transforming growth factor *β*/SMAD, and Hippo signaling pathways [17, 18]. The Wnt/*β*-catenin pathway is important in cell fate determination, and its activation and inhibition are essential for the formation of the cartilage and bone. There are many types of Wnt ligands, but their roles in cartilage differentiation require elucidation.

In this study, we explored the function and underlying mechanism of SCRG1 in chondrogenic differentiation of human UCMSCs *in vivo* and *in vitro*. Our findings should contribute to the prospective application of tissue engineering in regenerative medicine.

## 2. Materials and Methods

### 2.1. Cell Isolation and Culture of UCMSCs

This study was approved by Ethics Committee of the Yan'an Hospital Affiliated to Kunming Medical University (Certificate Number: 2008-001). Umbilical cords were obtained from healthy donors, who provided signed informed consent in advance. The UCMSCs were isolated and cultured according to our previous method [5, 19]. The UCMSCs were cultured in *α*-Minimum Essential Medium (*α*-MEM) supplemented with 10% fetal bovine serum, 1% penicillin-streptomycin, and 5 ng/mL basic fibroblast growth factor. When cell growth reached 80%–90% confluency, they were passaged by trypsinization. Cells at passages 4–6 were used in following experiments.

### 2.2. Flow Cytometry and Multilineage Differentiation

UCMSC surface markers were analyzed by flow cytometry. Cells were collected at a density of 1 × 10^6^ cells/mL, washed with phosphate-buffered saline (PBS), resuspended in staining buffer, and stained with antibodies (562245, BD, USA) against CD73, CD90, CD105, CD34, CD45, CD19, CD11b, and HLA-DR in the dark for 30 min. After that, the cells were washed and resuspended in PBS, then analyzed using a flow cytometer (FACSCanto™ II, BD, USA). Osteogenic, adipogenic, and chondrogenic differentiations were performed to evaluate the differentiation potential of UCMSCs. Osteogenic and adipogenic differentiation culture protocols follow the manufacturer's instructions. On day 21, calcium mineralization and lipid droplets were detected using Alizarin Red S and Oil Red O staining techniques, respectively. A microsphere culture system was used for chondrogenic differentiation. Cells were collected at a density of 2 × 10^7^ cells/mL, and a 10 *μ*L droplet was seeded into 12-well plates. After 2 h of cell adhesion, chondrogenesis-inducing medium (05-220-1B, BI, Israel) was added to the plates. The medium was replaced twice a week. On day 21, glycosaminoglycans were detected by Alcian Blue staining.

### 2.3. Gene Transfection by Lentiviral Particles

All lentiviral particles (knockdown and overexpression) were purchased from Genechem (Shanghai, China). Three short-hairpin RNAs (shRNAs) against SCRG1 were designed, and the most effective (see Table 1 for sequence information) was used for subsequent experiments. For viral transfection, UCMSCs were seeded in six-well plates at a density of 2 × 10^5^ cells/well. Virus-containing medium was added when the cells reached 30% confluence. After 12 h of viral transfection, the medium was replaced with complete medium. At 72 h after transfection, cells were selected with puromycin (2 *μ*g/mL). Cells were harvested for analysis or passaged at 80%–90% confluency. Gene expression of SCRG1 was quantified by qRT-PCR.

### 2.4. Total RNA Extraction and qRT-PCR

Total RNA was extracted from cells and microspheres using a Total RNA Super Extraction Kit (LS1040, Promega, USA), and total RNA was reverse transcribed into cDNA using a GoScript Reverse Transcription Mix (A2791, Promega, USA). We performed qRT-PCR using 1 *μ*g of cDNA with a SsoFast EvaGreen® Supermix Kit (1725201, Bio-Rad, USA) according to the manufacturer's instructions. The forward and reverse primers sequences are shown in Table 2. Each sample was analyzed in triplicate. Gene expression results were normalized to *β*-actin and presented as fold-change compared to the controls.

### 2.5. RNA Sequencing (RNA-seq) and Bioinformatics Analysis

RNA-seq was performed to identify which signaling pathway was activated by SCRG1 overexpression. The overexpression negative control group (OE-nc) and overexpression SCRG1 group (OE-SCRG1) were harvested, with three samples analyzed for each group. Total RNA was extracted using TRIzol® Reagent (Invitrogen, USA) and used as input material for cDNA library preparation, followed by sequencing using Illumina NovaSeq 6000. DEG analysis of the two groups was performed using the DESeq2 R package (v1.20.0). Gene Set Enrichment Analysis (GSEA) was applied to identify significant and consistent differences between the two biological states. Genes were ranked according to the degree of differential expression in the two samples. The predefined gene set was then tested to determine if the genes were enriched at the top or bottom of the list. The Kyoto Encyclopedia of Genes and Genomes (KEGG) data sets were analyzed using the local version of the GSEA tool (http://www.broadinstitute.org/gsea/index.jsp).

### 2.6. Alcian Blue and Safranin O/Fast Green Staining

Microspheres of UCMSCs were differentiated in chondrogenic medium for 21 days, then fixed in 4% paraformaldehyde (PFA) overnight. Next, samples were embedded in paraffin and cut into 5 *μ*m slices, which were deparaffinized, rehydrated, and stained with 1% Alcian Blue overnight at room temperature. After this, the slides were then rinsed three times using 0.1 N HCl and distilled water to neutralize acidity, then dehydrated with anhydrous ethanol, and sealed with neutral gum. Images were captured by a Nikon Eclipse 50i (Japan). For Safranin O/Fast Green staining, the slices were deparaffinized, rehydrated with PBS, and stained with Fast Green solution for 5 min. Afterwards, the slides were washed in water, incubated in Safranin O solution for 30 s, dehydrated with anhydrous ethanol twice, sealed with neutral resin, and imaged using Nikon Eclipse 50i (Japan).

### 2.7. Western Blot Analysis

The UCMSCs were lysed with radioimmunoprecipitation assay buffer supplemented with protease inhibitor. Protein concentration was determined using a BCA assay kit (Beyotime Biotechnology, China). The protein lysates (20–40 *μ*g) were separated by 10% sodium dodecyl sulfate-polyacrylamide gel electrophoresis (SDS-PAGE) and transferred to polyvinylidene fluoride membranes (PVDF), which were then blocked with 5% skim milk in tris-buffered saline with Tween 20 (TBST) at room temperature for 1 h, followed by incubation with antibodies against Wnt5a (1 : 1000; #2530S, Cell Signaling Technology, USA), *β*-catenin (1 : 1000; #8480 s, Cell Signaling Technology, USA), and GAPDH (1 : 5000; 60004-1-Ig, Proteintech, USA) overnight at 4°C. The membranes were then incubated with horseradish peroxidase- (HRP-) conjugated secondary antibodies (1 : 5000, SA00001-2, Proteintech, USA) at room temperature for 1 h. Protein bands were visualized using an enhanced chemiluminescence (ECL) kit (KF005, Affinity Biosciences, USA) and detected using a ChemiDoc™ Touch Imaging System (Bio-Rad, USA). Intensities of the bands were determined using ImageJ software (https://imagej.nih.gov/ij/) and normalized to GAPDH levels. To evaluate the effects of SCRG1 on Wnt5a and *β*-catenin expression in UCMSCs, the cells were treated with recombinant human SCRG1 (rhSCRG1, ab161421, Abcam, UK) at a concentration 450 ng/mL for 1 h.

### 2.8. Immunohistochemistry

For immunohistochemical (IHC) staining, the slices were deparaffinized in xylene and rehydrated in graded ethanol. Endogenous peroxidase activity was blocked with 3% H_2_O_2_ for 10 min. After blocking with 5% goat serum at room temperature for 1 h, slices were incubated overnight at 4°C with the following primary antibodies: SCRG1 (1 : 800; 14038-1-AP, Proteintech, USA), Col2a1 (1 : 400; 28459-1-AP, Proteintech, USA), and ACAN (1 : 800; 13880-1-AP-100 *μ*L, Proteintech, USA). The following day, the slices were incubated with streptavidin-HRP-conjugated secondary antibodies for 1 h, then visualized using a diaminobenzidine substrate. The slices were counterstained with hematoxylin, dehydrated, and sealed with neutral gum. The mean optical density (MOD) of positive immunostaining was analyzed using ImageJ Pro Plus v6.0. The protein expression of SCRG1 after lentiviral transfection was detected by IHC. The UCMSCs were seeded in 24-well plates. The coverslips were processed as described above for IHC staining for mouse anti-SCRG1 (1 : 200, 14038-1-AP, Proteintech, USA). To detect COL2A1 and ACAN expression in UCMSCs activated with recombinant human Wnt5a (rhWnt5a; ab204627, Abcam) and rhSCRG1, the cells were cultured in serum-free medium at 37°C for 4 h, then treated with rhWnt5a (300 ng/mL) and rhSCRG1 (450 ng/mL) at 37°C for 24 h. Next, IHC staining was performed with primary antibodies against COL2A1 (1 : 100) and ACAN (1 : 200) as described above.

### 2.9. Cartilage Defect Model and Implantation of UCMSCs *In Vivo*

All animal experiments were approved by the Ethics Committee of Yan'an Hospital Affiliated to Kunming Medical University (Certificate Number: 2020-090-01). To evaluate the effects of SCRG1 *in vivo*, we used 16-week-old male New Zealand white rabbits. A cartilage defect model was established via surgery at the femoral trochlear groove as follows. First, the rabbits were anesthetized with isoflurane, after which the knee joint capsule was opened layer by layer, and the patella was dislocated medially to expose the articular surface of the distal femur. Next, an electric drill with a 3.5 mm diameter was used to create a full thickness of cartilage defect on the nonstressed surface of the femoral trochlear groove, after which UCMSCs were added into the defect. There were three groups, i.e., blank control group (Blank), negative control lentiviral vector group (OE-nc), and overexpression of SCRG1 group (OE-SCRG1). For the blank group, only Matrigel (356231, Corning, USA) was administered to the defects. For the OE-nc and OE-SCRG1 groups, UCMSCs were transfected with negative control virus and overexpression of SCRG1 virus, respectively, then mixed with Matrigel on ice at a final concentration of 2 × 10^7^ cells/mL. This mixture was then applied to the defects. After the Matrigel solidified, the patella was repositioned, and the knee joint capsule and skin were closed layer-by-layer using appropriate sutures. All rabbits were injected intramuscularly with penicillin for three consecutive days to prevent infection and were allowed to move freely in their cage.

### 2.10. Pathological Analysis of Repaired Tissues

At 12 weeks after surgery, the rabbits were sacrificed under deep anesthesia, and femoral heads were isolated for imaging and macroscopic evaluation according to the guidelines of the International Cartilage Repair Society (ICRS) scoring system [20]. Subsequently, each femoral head was fixed in 4% PFA for 48 h and decalcified for 3 weeks. Next, samples were embedded in paraffin and sectioned into 5 *μ*m slices and stained with hematoxylin and eosin (H&E), Alcian Blue, and Safranin O. Regenerated tissue was analyzed according to a modified ICRS histological scoring system, and each sample was evaluated by three pathologists blind to slide information.

### 2.11. Statistical Analysis

Statistical analysis was performed with GraphPad Prism 8 (GraphPad Software, Inc., San Diego, CA, USA). All data are presented as mean values with standard deviations (mean ± SD). Differences were evaluated by Student's *t*-test or one-way analysis of variance (ANOVA). A *P* value of <0.05 was considered statistically significant.

## 3. Results

### 3.1. Identification and Differentiation of UCMSCs

**Figure 1:**
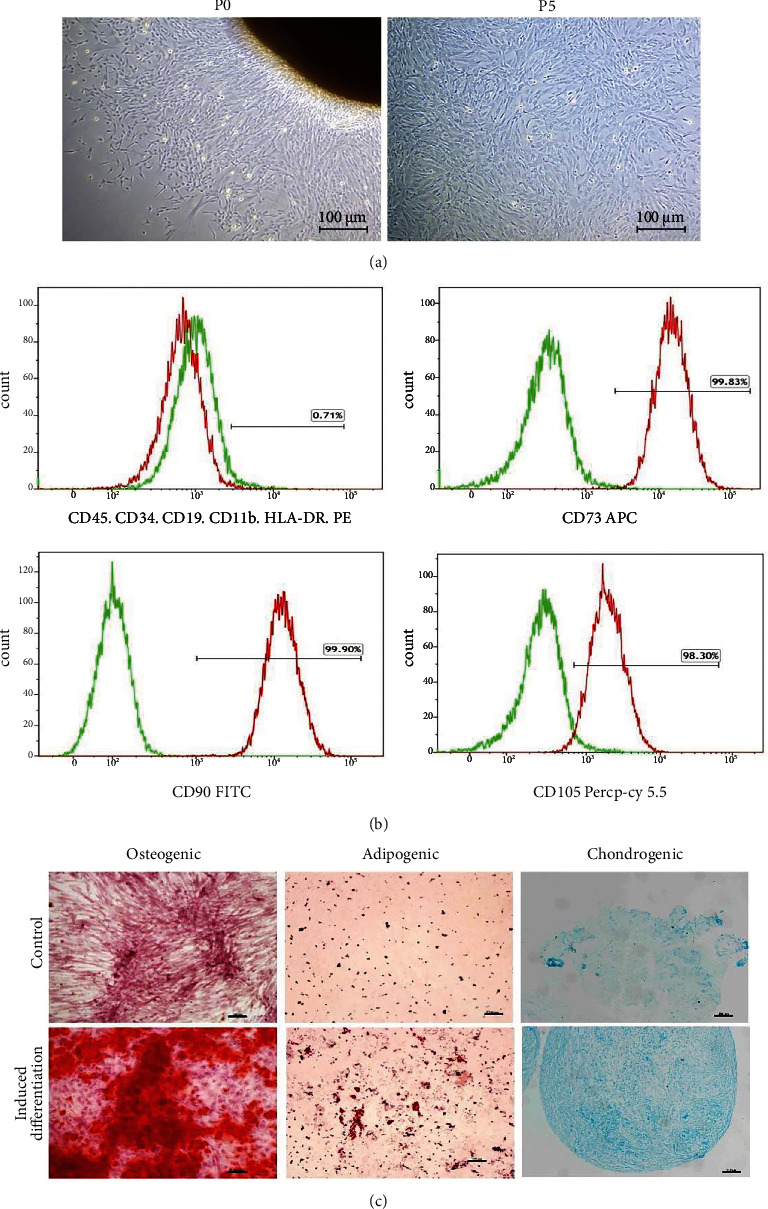
Isolation and identification of UCMSCs. (a) Photographs of UCMSCs at passage 0 (P0) and P5, showing spindle-shaped UCMSCs. Scale bar = 100 *μ*m. (b) Flow cytometry results. Green line represents isotype control, and red line represents UCMSCs. UCMSCs with positive CD73, CD90, and CD105 expression and negative CD45, CD34, CD19, CD11b, and HLA-DR expression. (c) Alizarin Red S, Oil Red O, and Alcian Blue staining. Results showed that UCMSCs differentiated into osteocytes, adipocytes, and chondrocytes. Scale bar = 100 *μ*m.

After 7 days, many spindle cells were observed in the primary culture (Figure 1(a)). Two weeks later, the cells reached 80% confluency, at which time they were trypsinized for further passage. Cells at passage 5 (Figure 1(a)) were collected for flow cytometry analysis. Results showed that cells expressed CD73 (positive rate: 99.83%), CD90 (positive rate: 99.90%), and CD105 (positive rate: 98.30%) but were negative for CD45, CD34, CD19, CD11b, and HLA-DR (Figure 1(b)), consistent with the identification standards for MSCs [21]. These results indicated that the isolated cells were UCMSCs. Functional characterization of the UCMSCs was further validated by histological staining. Result showed that the UCMSCs could differentiate into osteogenic, adipogenic, and chondrogenic lineages, as illustrated by the Alizarin Red S, Oil red O, and Alcian Blue staining, respectively. These findings indicated that the UCMSCs possessed multilineage differentiation potential (Figure 1(c)).

### 3.2. SCRG1 Was Upregulated during Chondrogenic Differentiation of UCMSCs

**Figure 2:**
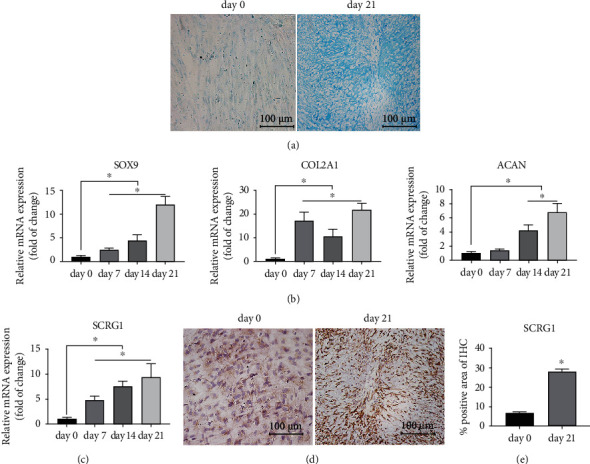
SCRG1 was upregulated during chondrogenic differentiation. (a) Alcian Blue staining of UCMSCs chondrogenic differentiation at day 0 and day 21; results showed accumulation of glycosaminoglycans at day 21 after chondrogenic differentiation, indicating chondrogenesis of UCMSCs. Scale bar = 100 *μ*m. (b) mRNA expression levels of chondrogenic genes (*SOX9*, *COL2A1*, and *ACAN*) were upregulated during induction. (c) mRNA expression level of SCRG1 was upregulated during induction. (d, e) Protein level of SCRG1 was upregulated after 21 days chondrogenic differentiation, as detected by IHC. Scale bar = 100 *μ*m. Experiments were repeated three times; ^∗^*P* < 0.05 (*t*-test).

To evaluate the SCRG1 expression levels during chondrogenic differentiation of UCMSCs, the cells were cultured in a chondrogenesis-inducing medium using a microsphere culture system for 21 days. First, to validate chondrogenic differentiation of UCMSCs, Alcian Blue staining was performed to evaluate glycosaminoglycans in the extracellular matrix secreted by the chondrocytes, with qRT-PCR then used to quantify the expression levels of chondrocyte markers. Results showed the accumulation of glycosaminoglycans in the extracellular matrix (Figure 2(a)) at 21 days, and the expression levels of chondrocyte markers were significantly increased during chondrogenic differentiation (Figure 2(b)). In addition, qRT-PCR was used to measure the expression level of *SCRG1* during chondrogenesis, which showed a continuous and significant increase during chondrogenic differentiation (Figure 2(c)). We also analyzed the protein expression levels of SCRG1 after 21 days of induction by IHC staining. Result showed a significant increase in SCRG1-protein expression during chondrogenic differentiation (Figures 2(d) and 2(e)), consistent with the qRT-PCR findings.

### 3.3. SCRG1 Knockdown Suppressed Chondrogenic Differentiation of UCMSCs

**Figure 3:**
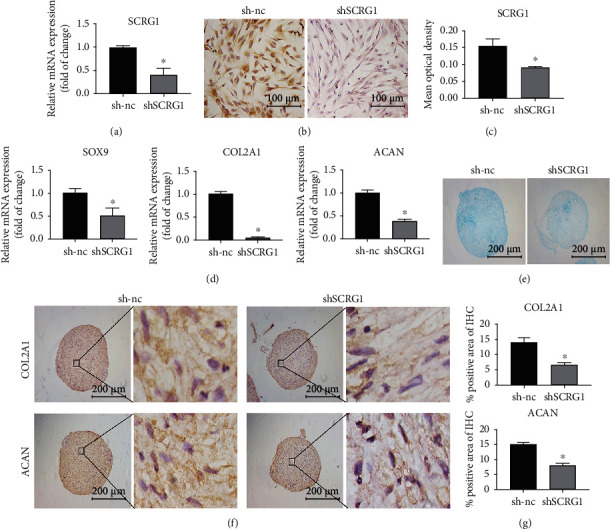
SCRG1 knockdown inhibited chondrogenic differentiation potential of UCMSCs. (a–c) mRNA and protein expression levels of SCRG1 were decreased after transfection with *SCRG1* shRNA, detected by qRT-PCR and IHC. Scale bar = 100 *μ*m. (d) *SOX9*, *COL2A1*, and *ACAN* mRNA levels decreased following SCRG1 knockdown. (e) Alcian Blue staining at day 21. Results showed that glycosaminoglycan synthesis decreased significantly after SCRG1 knockdown. (f, g) Protein levels of COL2A1 and ACAN detected by IHC at day 21 decreased after SCRG1 knockdown. Scale bar = 200 *μ*m. Experiments were repeated three times; ^∗^*P* < 0.05 (*t*-test).

To examine the effects of SCRG1 on chondrogenic differentiation of UCMSCs, we used lentiviral vectors expressing shRNA targeting SCRG1 in UCMSCs. The qRT-PCR results showed that *SCRG1* expression significantly decreased after transfection with *SCRG1* shRNA (Figure 3(a)). The protein expression level of SCRG1 also decreased after transfection with *SCRG1* shRNA (Figures 3(b) and 3(c)). The transcription factor SOX9 plays an important role in chondrogenic differentiation, and the chondrocyte extracellular matrix is rich in collagens and proteoglycans, with COL2A1 and ACAN being the most representative [11]. After SCRG1 knockdown, the mRNA expression levels of *SOX9*, *COL2A1*, and *ACAN* decreased significantly, indicating that SCRG1 may be involved in chondrogenic differentiation (Figure 3(d)). Alcian Blue staining showed that the synthesis of proteoglycans was inhibited after *SCRG1* knockdown (Figure 3(e)). Furthermore, IHC staining showed that the expression levels of COL2A1 and ACAN were reduced following *SCRG1* knockdown (Figures 3(f) and 3(g)).

### 3.4. SCRG1 Promoted Chondrogenic Differentiation of UCMSCs *In Vitro*

**Figure 4:**
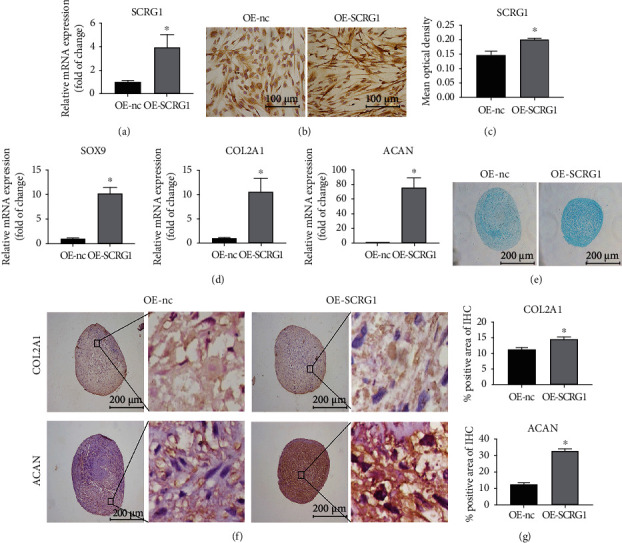
SCRG1 overexpression promoted chondrogenic differentiation of UCMSCs. (a–c) mRNA and protein levels of SCRG1 were detected by qRT-PCR and IHC. Results showed that mRNA and protein levels of SCRG1 increased after overexpression of SCRG1. Scale bar = 100 *μ*m. (d) *SOX9*, *COL2A1*, and *ACAN* mRNA levels increased after overexpression of SCRG1. (e) Alcian Blue staining showed an increase in glycosaminoglycans at day 21 after overexpression of SCRG1. Scale bar = 200 *μ*m. (f, g) Protein levels of COL2A1 and ACAN detected by IHC at day 21 increased after SCRG1 knockdown. Scale bar = 200 *μ*m. Experiments were repeated three times; ^∗^*P* < 0.05 (*t*-test).

We next overexpressed SCRG1 in the UCMSCs using a lentiviral vector. The qRT-PCR and IHC results showed that SCRG1 expression increased following transfection (Figures 4(a)–4(c)). Furthermore, qRT-PCR analysis of cells overexpressing SCRG1 (OE-SCRG1) showed that the expression levels of chondrogenic genes *SOX9*, *COL2A1*, and *ACAN* increased significantly compared with that in the negative control group (OE-nc) (Figure 4(d)). Alcian Blue staining showed that SCRG1 overexpression increased glycosaminoglycans synthesis (Figure 4(e)). Similarly, IHC demonstrated that SCRG1 overexpression increased the protein expression levels of COL2A1 and ACAN (Figures 4(f) and 4(g)). Thus, SCRG1 knockdown inhibited chondrogenic differentiation of UCMSCs, whereas SCRG1 overexpression promoted chondrogenic differentiation.

### 3.5. SCRG1 Promoted Chondrogenic Differentiation of UCMSCs through Wnt5a Signaling Pathway

**Figure 5:**
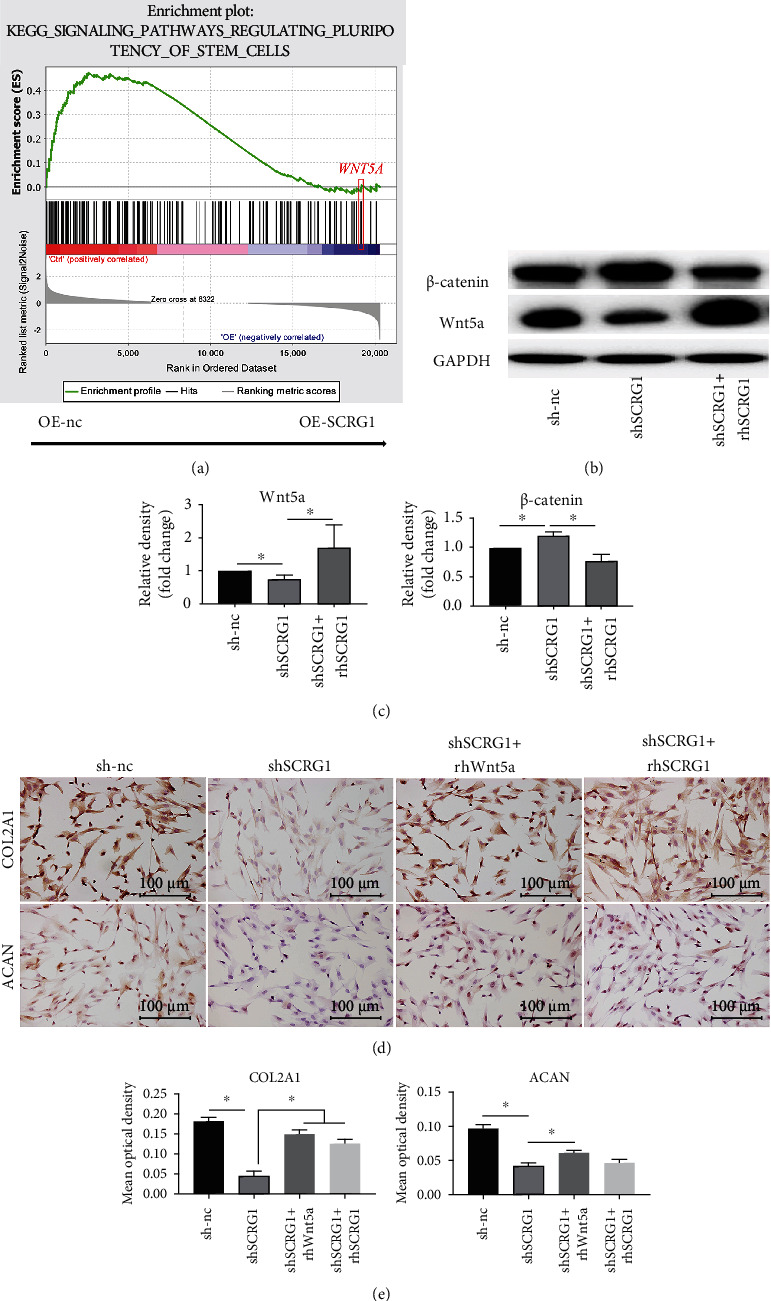
SCRG1 regulated chondrogenesis of UCMSCs through Wnt5a signaling pathway. (a) Regulating pluripotency of stem cell signaling pathway was the most significantly enriched KEGG pathway based on GSEA, wnt5a was negatively correlated with this pathway after SCRG1 overexpression. (b, c) Western blot analysis showed that Wnt5a decreased after shSCRG1 transfection and was restored after administration of rhSCRG1 (450 ng/mL); protein levels of *β*-catenin exhibited the opposite trend to Wnt5a. (d, e) Protein levels of COL2A1 and ACAN were detected in UCMSCs by IHC. The results showed that the protein levels of COL2A1 and ACAN were reduced following SCRG1 knockdown and restored after treated with rhWnt5a (300 ng/mL) and rhSCRG1 (450 ng/mL) protein at 24 h. Scale bar = 100 *μ*m. Experiments were repeated three times, ^∗^*P* < 0.05 (*t*-test).

To determine the mechanism underlying the promotion effects of SCRG1 on chondrogenesis in UCMSCs, we performed RNA-seq to identify the DEGs between the OE-nc and OE-SCRG1 groups. Based on this result, we performed KEGG pathway enrichment analysis using GSEA and found the most significantly enriched pathways was “KO 04550” (signaling pathways regulating pluripotency of stem cells) (Figure S1). In this signaling pathway, we noticed that Wnt5a was top enriched in the bottom of the genes list. Results showed Wnt5a was negatively correlated to this pathway when SCRG1 overexpression (Figure 5(a), S2). Wnt/*β*-catenin signaling plays an important role in cartilage differentiation [22]. Thus, we hypothesized that SCRG1 regulates cartilage differentiation through the Wnt5a signaling pathway. Based on western blot analysis, after knockdown of SCRG1 in the UCMSCs, Wnt5a was significantly reduced and *β*-catenin was significantly increased. However, after administration of rhSCRG1, the protein expression level of Wnt5a recovered and *β*-catenin decreased (Figures 5(b) and 5(c)). SCRG1 was positively related to Wnt5a and negatively related to *β*-catenin, suggesting that Wnt5a may promote chondrogenic differentiation by suppressing the canonical Wnt/*β*-catenin signaling pathway. To further determine the effects of Wnt5a on chondrogenesis in UCMSCs, the cells were treated with rhWnt5a and rhSCRG1protein after SCRG1 knockdown, then subjected to cellular IHC staining. Results indicated that the protein expression levels of COL2A1 and ACAN were reduced following SCRG1 knockdown and restored after treated with rhWnt5a and rhSCRG1protein at 24 h, suggesting that SCRG1 promoted chondrogenesis of UCMSCs by Wnt5a (Figures 5(d) and 5(e)).

### 3.6. UCMSC^OE-SCRG1^ Promoted Cartilage Regeneration *In Vivo*

**Figure 6:**
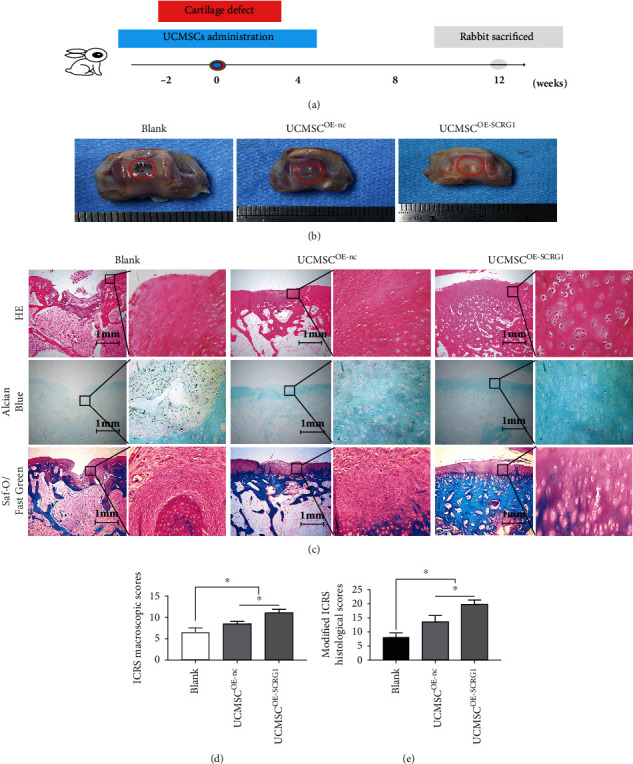
UCMSC^OE-SCRG1^ promoted cartilage defects repair *in vivo*. (a) Schematic of cartilage defect model and UCMSC administration. (b) Photographs of cartilage defects at 12 weeks after UCMSC administration. Obvious defects were observed in the blank group, whereas the surface was smooth and regenerated tissue was tightly connected to surrounding cartilage in the OE-SCRG1 group. (c) H&E, Alcian Blue, and Safranin O/Fast Green staining at 12 weeks after surgery. Results showed that defects in the blank group contained necrotic and fibrous tissue, while the OE-SCRG1 group showed considerable chondrocyte regeneration and tissue morphologies similar to normal surrounding cartilage. (d) ICRS macroscopic scores at 12 weeks after surgery. Scores in the OE-SCRG1group were significantly higher than those in the OE-nc group (*P* < 0.05). (e) Modified ICRS histological scores at 12 weeks after surgery. Scores were significantly higher in the OE-SCRG1 group than in the OE-nc group (*P* < 0.05). Scale bar = 200 *μ*m. *N* = 6; ^∗^*P* < 0.05 (*t*-test).

To validate the effects of SCRG1 on cartilage regeneration in UCMSCs *in vivo*, we transplanted UCMSCs into a cartilage defect model (Figure 6(a)). At 12 weeks after transplantation, the rabbits were euthanized, and trochlear osteochondral samples were harvested for macroscopic and histological evaluation. For macroscopic evaluation, obvious defects were observed in the control group, and a few transparent tissues were visible at the base of the defects. In the OE-nc group, the defects contained transparent tissues, and small, scattered fissures or cracks were visible. The regenerated tissue was almost completely integrated with the surrounding cartilage, and the defect surface was slightly uneven. In the OE-SCRG1 group, the defect surface was smooth and regenerated tissues were tightly connected to the surrounding cartilage, with no fissures or cracks detected (Figure 6(b)). The ICRS scores (Figure 6(d)) were significantly higher in the OE-SCRG1 group than in the OE-nc and control groups (*P* < 0.05). For histological evaluation, H&E staining showed that defects in the control group were filled with necrotic and fibrous tissue, while Alcian Blue and Safranin O/Fast Green staining revealed no glycosaminoglycan and chondrocyte regeneration in the defects. In the OE-nc group, H&E staining showed that the defects contained cells and fibrous tissue, while Alcian Blue staining identified some glycosaminoglycan expression in the regenerated tissue, and Safranin O/Fast Green staining indicated the occurrence of chondrocyte regeneration in the defects. In the OE-SCRG1 group, H&E staining showed considerable cell proliferation in the defects, with similar cell morphology as the surrounding normal cartilage, as well as few fibrous tissues on the defect surface. Alcian Blue staining identified a large amount of glycosaminoglycans in the defects and Safranin O/Fast Green staining indicated that a large number of chondrocytes were generated, and the generated tissue was the same as the surrounding cartilage tissue (Figure 6(c)). Modified ICRS histological scoring (Figure 6(e)) also showed that defect repair in the OE-SCRG1 group was significantly higher than that in the OE-nc and control groups (*P* < 0.05). In summary, these results suggest that SCRG1 overexpression promotes chondrogenic differentiation of UCMSCs in vivo.

## 4. Discussion

Chondrogenic differentiation is one of the most important abilities of MSCs. Compared with other types of MSCs, UCMSCs have many advantages, such as powerful differentiation ability, noninvasive collection, no tumorigenicity, and low immunogenicity. Thus, UCMSCs are an ideal source of seed cells for cartilage tissue engineering. However, the underlying molecular mechanism by which UCMSCs generate cartilage requires further research. In this study, we successfully isolated UCMSCs and found that SCRG1 promoted chondrogenic differentiation of UCMSCs *in vitro* and *in vivo* by inhibited canonical Wnt/*β*-catenin signaling pathway through Wnt5a.

Due to the weak regeneration ability of cartilage, there is an increasing demand for advanced approaches or new targets to improve the precision and efficacy of cartilage defect repair. Directed differentiation of MSCs into chondrocytes is a promising direction for regenerative medicine and has been widely used in the regeneration of cartilage defects [23]. Although a variety of methods have been applied to promote chondrogenic differentiation, such as growth factors, exosomes, long noncoding RNAs (lncRNA), and microRNAs [24, 25], further research is required to identify an optimal method. While SCRG1 is expressed in many tissues of the human body at tissue-specific levels, its functions remain poorly known. Here, to examine the function of SCRG1 in UCMSC chondrogenic differentiation, we used lentiviral vectors to inhibit or overexpress *SCRG1*. Results show that SCRG1 positively regulated the expression of chondrogenic genes, including *SOX9*, *COL2A1*, and *ACAN*, similar to previous research [9, 25], indicating that SCRG1 promoted chondrogenic differentiation of UCMSCs *in vitro*. Chondrogenic and osteogenic differentiation occurs in opposite directions in stem cells [26, 27]. Various transcription factors, such as Wnt10b, SOX9, and RUNX2 [22, 28, 29], are upregulated or downregulated to determine the cell fate of MSCs during chondrogenic and osteogenic differentiation. Previous studies have revealed that SCRG1 is highly expressed during cartilage differentiation and dramatically reduced during osteogenic differentiation [30, 31]. Similarly, our results indicated that overexpression of SCRG1 promoted the differentiation of UCMSCs into cartilage and may be a key factor in chondrogenic commitment of UCMSCs.

We established a full-thickness cartilage defect model in the knee joints of New Zealand white rabbits, a commonly used animal model for cartilage repair experiments. Unlike other models, we did not use a triple-combination graft composed of cells, scaffolds, and inducers [7, 32, 33]. Instead, we used growth factor-reduced Matrigel as a scaffold. As the Matrigel is liquid on ice and thoroughly solidified at 37°C, we mixed the UCMSCs with Matrigel on ice and then transplanted the mixture into the defects. At 12 weeks after transplantation, the defects contained regenerated tissue but no Matrigel, suggesting it was likely absorbed. Thus, we consider Matrigel to be a competent scaffold for MSC transplantation. We also evaluated cartilage repair using the standard ICRS macroscopic scoring system and a modified ICRS histological scoring system, with higher scores indicating better repair. Based on our results, the OE-SCRG1 group achieved the highest scores. For histological analysis, H&E staining showed that the OE-SCRG1 group defects were filled with regenerated cells and did not contain necrotic or fibrous tissue. Alcian Blue staining showed that many glycosaminoglycans were generated, and Safranin O/Fast Green staining showed that the regenerated cells in the defects were chondrocytes. However, although our observation of regenerated chondrocytes in the defects indicates that SCRG1 promoted cartilage regeneration *in vivo*, whether the chondrocytes arose from endogenous rabbit cells or differentiated from UCMSCs requires further study.

We also performed RNA-seq and bioinformatics analysis and found that SCRG1 promoted chondrogenic differentiation through the Wnt5a signaling pathway. Based on GSEA, we analyzed KEGG pathway enrichment. Results showed that the most significantly enriched pathway was related to “regulating pluripotency of stem cells” (KO 04550) (Figure S1). This pathway is involved in cell fate commitment. When SCRG1 was overexpressed, UCMSCs start to differentiate and lose their pluripotency. Our results showed that SCRG1 overexpressed promoted chondrogenic differentiation of UCMSCs and UCMSCs might be differentiated into chondrocyte. Furthermore, wnt5a was top enriched gene in pathway KO 04550 after SCRG1 overexpression (Figure S2). These findings suggest that wnt5a is related to cell differentiation, and SCRG1 overexpression promotes chondrogenic differentiation of UCMSCs. Thus, we believe that Wnt5a is a key factor of chondrogenic differentiation of UCMSCs. After knockdown of SCRG1 in the UCMSCs, Wnt5a was significantly reduced and *β*-catenin was significantly increased. However, after administration of rhSCRG1, the protein expression level of Wnt5a recovered and *β*-catenin decreased (Figure 5(b)). We also found that the protein expression levels of COL2A1 and ACAN were reduced following SCRG1 knockdown and restored after treatment with rhWnt5a and rhSCRG1protein at 24 h (Figure 5(d)). Thus, we believe that Wnt5a is a key factor of chondrogenic differentiation of UCMSCs.Wnt5a, a ligand in the Wnt signaling pathway, exerts cell type-specific functions by activating canonical Wnt/*β*-catenin pathway or inhibiting canonical Wnt/*β*-catenin pathway [34]. In chondrogenic differentiation of MSC, Wnt5a inhibits the canonical Wnt/*β*-catenin signaling pathway and promotes MSCs differentiate into the cartilage [22, 35], consistent with the results of our study. To examine whether SCRG1 interacts with Wnt5a, we performed reverse coimmunoprecipitation (co-IP) experiments. Unfortunately, our results showed that SCRG1 did not directly connect with Wnt5a (Table S1). We also performed protein-protein interaction (PPI) analysis using interactions from the GeneMANIA PPI database (https://genemania.org/) between enriched Wnt5a and SCRG1, and we found in previous studies (Figure S3) that SCRG1 and Wnt5a could indirectly connect through FZD1 and WIF1 [36, 37]. Based on the above results, we speculated that SCRG1 promotes chondrogenic differentiation through the Wnt5a signaling pathway. The canonical Wnt/*β*-catenin signaling pathway is closely related to osteogenic and chondrogenic differentiation of stem cells [38]. Previous research has shown that activating the canonical Wnt/*β*-catenin signaling pathway can lead to osteogenic differentiation of MSCs [39], whereas its inhibition can promote chondrogenic differentiation of MSCs [40]. Activating the pathway can reduce *β*-catenin degradation, giving rise to osteogenic differentiation, while inhibiting the pathway can increase *β*-catenin degradation, giving rise to chondrogenic differentiation [41]. Wnt5a, a ligand in the Wnt signaling pathway, exerts cell type-specific functions by binding to its receptors. Specifically, Wnt5a can inhibit the canonical Wnt/*β*-catenin signaling pathway and promote differentiation of MSCs into cartilage [22, 35], consistent with the results of our study. On the other hand, previous research has reported that Wnt5a can activate canonical Wnt/*β*-catenin signaling to inhibit differentiation of fibro/adipogenic progenitors into adipocytes [42]. Thus, Wnt5a may promote cartilage formation while inhibiting the differentiation of stem cells into other cell types. However, this mechanism requires further study.

## 5. Conclusions

This study demonstrated that SCRG1 promotes chondrogenic differentiation of UCMSCs via inhibited canonical Wnt/*β*-catenin signaling pathway through Wnt5a, thus providing a potential new target for high-efficacy cartilage regeneration and productive chondrogenic differentiation.

## Figures and Tables

**Table 1:** Information on shRNA targeting SCRG1.

No.	5′	STEM	Loop	STEM	3′
SCRG1-RNAi(90310-1)-a	Ccgg	ctTTGGACCAAAGATCTCTTT	CTCGAG	AAAGAGATCTTTGGTCCAAAG	TTTTTg
SCRG1-RNAi(90310-1)-b	aattcaaaaa	ctTTGGACCAAAGATCTCTTT	CTCGAG	AAAGAGATCTTTGGTCCAAAG	

**Table 2:** Primer sequences used in this study.

Gene	Forward primer	Reverse primer
SCRG1	5′-TCCTCTGAGCATCTTCGACC-3′	5′-CCCTTCGGTGCTGTGTAGTC-3′
SOX9	5′-CAAGAAAGACCACCCCGATTACA-3′	5′-ACCCTGAGATTGCCCAGAGTGCT-3′
COL2A1	5′-TGGATGCCACACTCAAGTCC-3′	5′-CACTCAGGGTGGCAGAGTTT-3′
ACAN	5′-ACTTCCGCTGGTCAGATGGA-3′	5′-TCTCGTGCCAGATCATCACC-3′
*β*-Actin	5′-CCTTCCTGGGCATGGAGTC-3′	5′-TGATCTTCATTGTGCTGGGTG-3′

## Data Availability

All data will be available on reasonable request.

## Supplementary Materials

Supplementary Materials Figure S1: top enriched KEGG pathway based on GSEA analysis. To explore the underlying mechanism, we performed RNA-seq and bioinformatics analysis and found that SCRG1 promoted chondrogenic differentiation through the Wnt5a signaling pathway. Based on GSEA, we analyzed KEGG pathway enrichment. Results showed that the most significantly enriched pathway was related to “regulating pluripotency of stem cells” (KO 04550). Figure S2: top enriched genes in “regulating pluripotency of stem cells” pathway. Wnt5a was top enriched genes and negatively correlated with this pathway after SCRG1 overexpression. These findings suggest that wnt5a is related to cell differentiation, and SCRG1 overexpression promotes chondrogenic differentiation of UCMSCs. Figure S3: SCRG1-related molecular networks, analyzed by genemania. We performed protein-protein interaction (PPI) analysis using interactions from the GeneMANIA PPI database (https://genemania.org/) between enriched Wnt5a and SCRG1, and we found in previous study (Figure S3) that SCRG1 and Wnt5a could indirectly connected through FZD1 and WIF1. Table S1: the protein list of Wnt5a antibody pulled down. To examine whether SCRG1 interacts with Wnt5a, we performed reverse coimmunoprecipitation (co-IP) experiments (as there is no commercial antibody against SCRG1 for western blot and co-IP analysis). Our results showed that SCRG1 did not directly connect with Wnt5a.
