# Supplementary material for: Scrapie-Responsive Gene 1 Promotes Chondrogenic Differentiation of Umbilical Cord Mesenchymal Stem Cells via Wnt5a
Source: Stem Cells Int. 2022 Jan 27;2022:9124277. doi: 10.1155/2022/9124277 (PMC8813292; doi:10.1155/2022/9124277)
Supplement: Supplementary Materials — Figure S1: top enriched KEGG pathway based on GSEA analysis. To explore the underlying mechanism, we performed RNA-seq and bioinformatics analysis and found that SCRG1 promoted chondrogenic differentiation through the Wnt5a signaling pathway. Based on GSEA, we analyzed KEGG pathway enrichment. Results showed that the most significantly enriched pathway was related to “regulating pluripotency of stem cells” (KO 04550). Figure S2: top enriched genes in “regulating pluripotency of stem cells” pathway. Wnt5a was top enriched genes and negatively correlated with this pathway after SCRG1 overexpression. These findings suggest that wnt5a is related to cell differentiation, and SCRG1 overexpression promotes chondrogenic differentiation of UCMSCs. Figure S3: SCRG1-related molecular networks, analyzed by genemania. We performed protein-protein interaction (PPI) analysis using interactions from the GeneMANIA PPI database (https://genemania.org/) between enriched Wnt5a and SCRG1, and we found in previous study (Figure S3) that SCRG1 and Wnt5a could indirectly connected through FZD1 and WIF1. Table S1: the protein list of Wnt5a antibody pulled down. To examine whether SCRG1 interacts with Wnt5a, we performed reverse coimmunoprecipitation (co-IP) experiments (as there is no commercial antibody against SCRG1 for western blot and co-IP analysis). Our results showed that SCRG1 did not directly connect with Wnt5a. [file 9124277.f1.pdf]

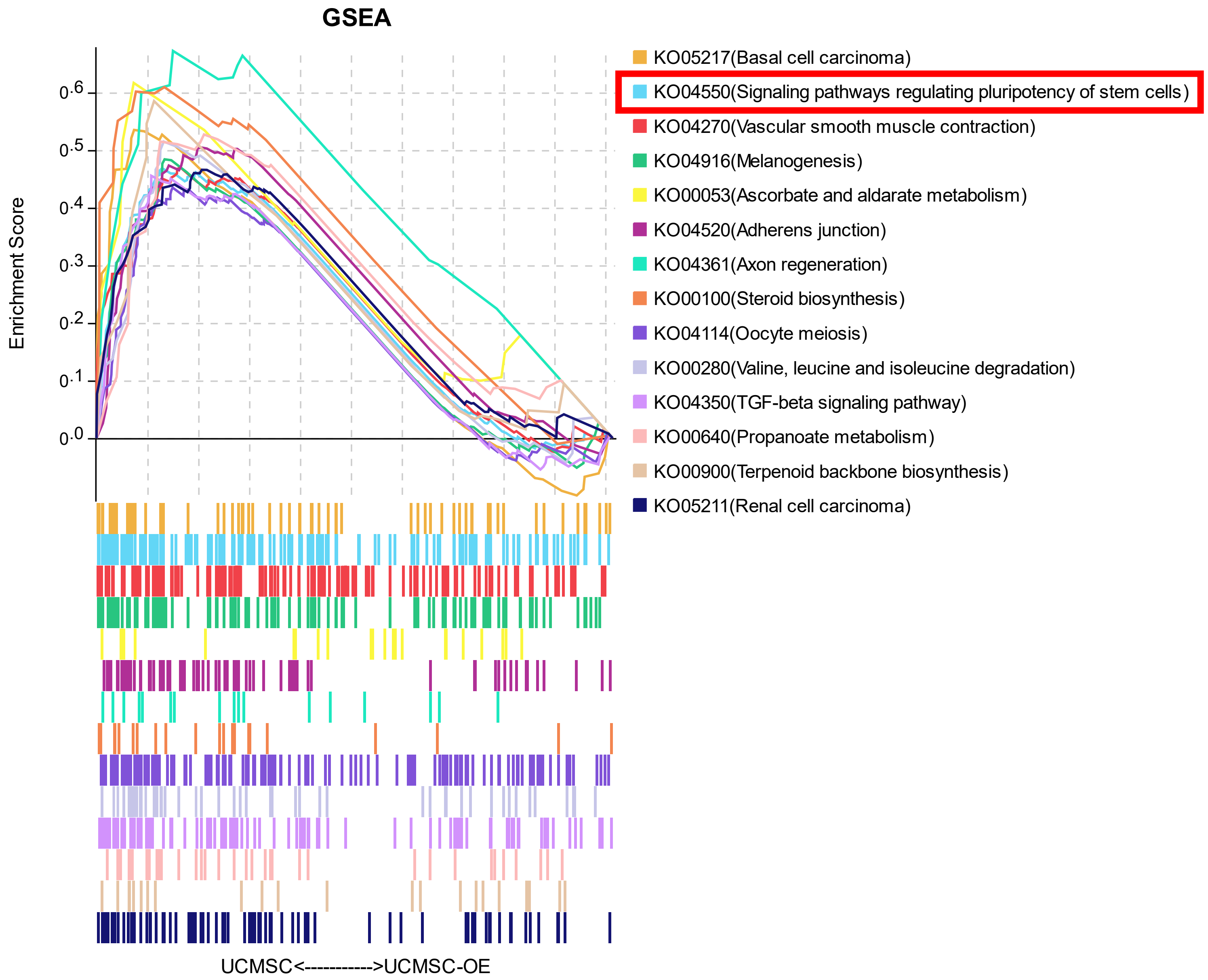

Figure S1. Top enriched KEGG pathway based on GSEA analysis

Figure S1. Top enriched KEGG pathway based on GSEA analysis

To explore the underlying mechanism, we performed RNA-seq and bioinformatics analysis, and found that SCRG1 promoted chondrogenic differentiation through the Wnt5a signaling pathway. Based on GSEA, we analyzed KEGG pathway enrichment. Results showed that the most significantly enriched pathway was related to “regulating pluripotency of stem cells” (KO 04550).

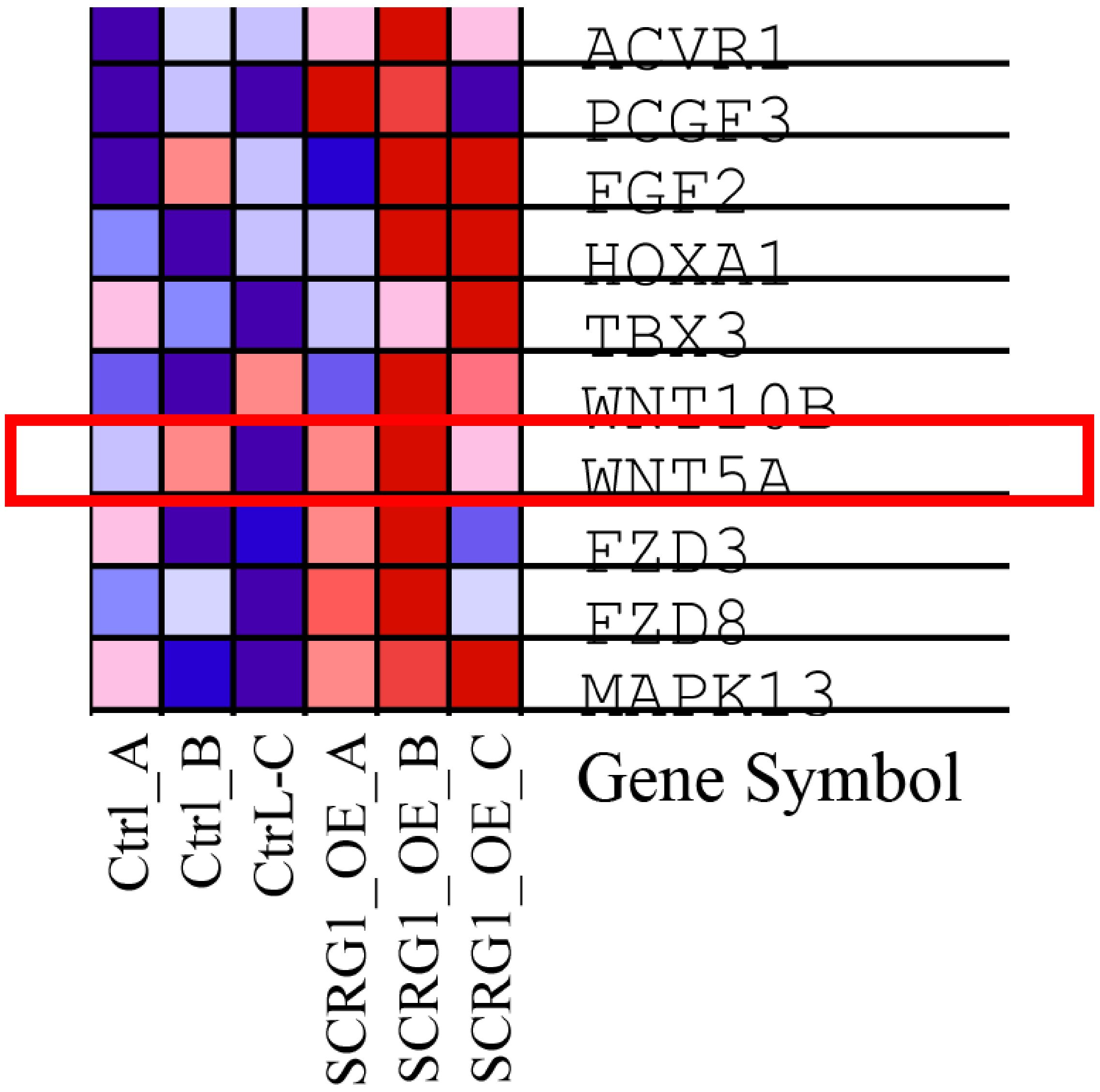

Figure S2. Top enriched genes in “regulating pluripotency of stem cells” pathway

Figure S2. Top enriched genes in “regulating pluripotency of stem cells” pathway

Wnt5a was top enriched genes and negatively correlated with this pathway after SCRG1 overexpression. These findings suggest that wnt5a is related to cell differentiation, and SCRG1 overexpression promotes chondrogenic differentiation of UCMSCs.

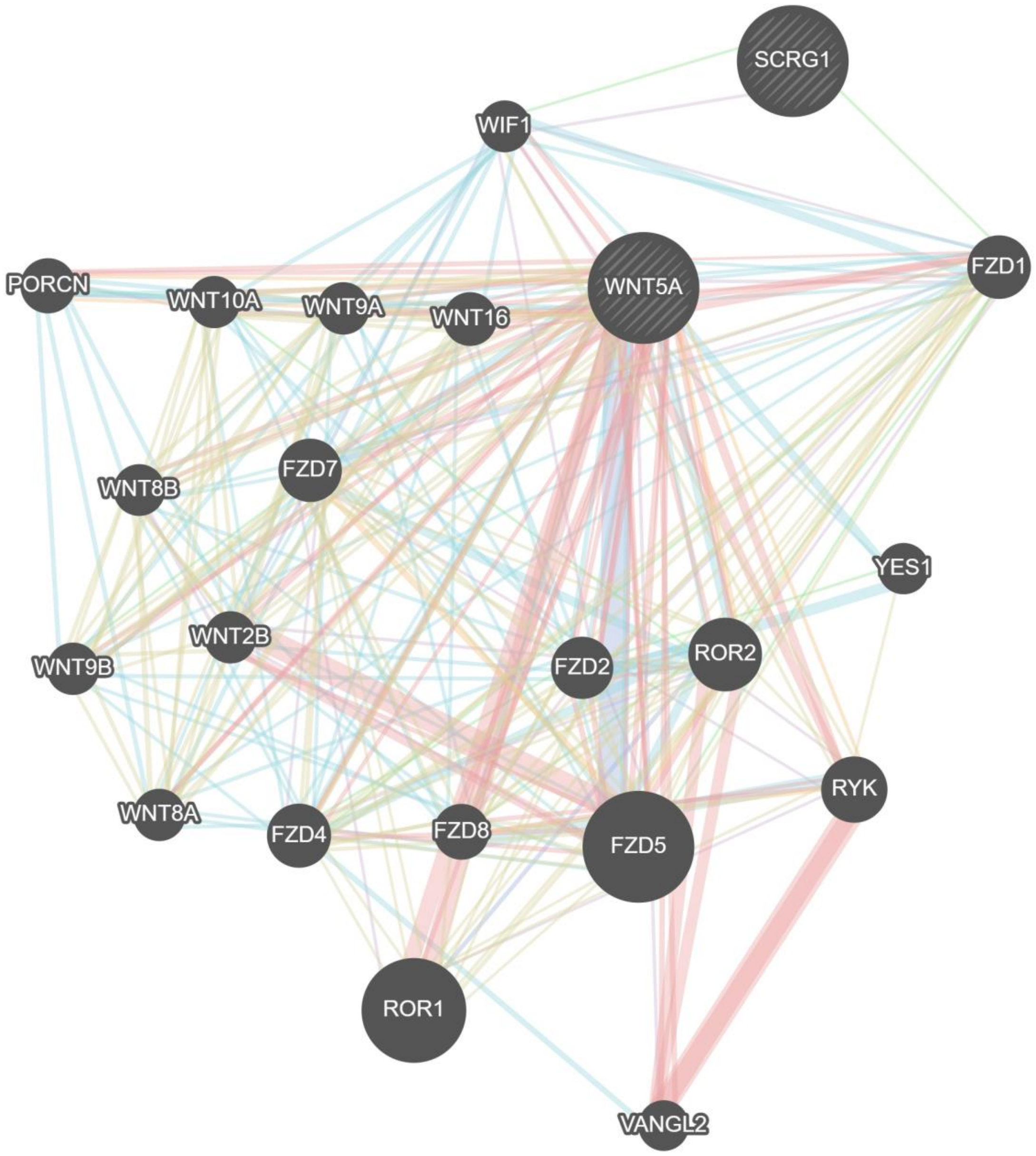

Figure S3: SCRG1 related molecular networks, analyzed by genemania

Figure S3: SCRG1 related molecular networks, analyzed by genemania

We performed protein-protein interaction (PPI) analysis using interactions from the GeneMANIA PPI database (https://genemania.org/) between enriched Wnt5a and SCRG1, and We found in previous study (Figure S3), SCRG1 and Wnt5a could indirectly connected through FZD1 and WIF1.

Gene symbols							
MYH9	VIM	DSP	HRNR	PLEC	MYH10	ACTG1	KPRP
HSPA5	P4HB	DSG1	LEPRE1	MYL9	ALB	TPM1	JUP
MYO1C	ANXA2	GAPDH	DCD	FLG2	MYL6	ACTA2	ACTG2
DSC1	FLG	CRTAP	AZGP1	S100A7	EEF1A1	PKP1	TUBA
S100A9	TRIM21	PIP	TXN	ALDOA	CALML5	MYH14	ACTB
FABP5	S100A8	GGCT	SERPINH1	SBSN	CLTC	CAT	FN1
RPS18	SERPINB3	HIST1H4A	SPTBN1	MS4A10	XP32	TUBB	FANCE
KIF18B	RPL13	NWD2	SPRR2B	ACT	CASP14	HSPA6	MYL12B
ZNF840P	FBXO3	TLN2					

Table S1: the protein list of Wnt5a antibody pulled down

Table S1: the protein list of Wnt5a antibody pulled down

To examine whether SCRG1 interacts with Wnt5a, we performed reverse co-immunoprecipitation (co-IP) experiments (as there is no commercial antibody against SCRG1 for western blot and co-IP analysis). Our results showed that SCRG1 did not directly connect with Wnt5a.
